# Association of *ALDH2* rs671 Polymorphism with chronic kidney disease incidence in a population-based Korean cohort

**DOI:** 10.1038/s41598-026-43186-4

**Published:** 2026-03-15

**Authors:** Hyun Jin Lee, Jaekyung Noh, Seunghwan Jeong, Heeyeon Lee, Haekyung Lee, Hyoungnae Kim, Jin Seok Jeon, Hyunjin Noh, Soon Hyo Kwon

**Affiliations:** 1 Division of Nephrology, Department of Internal Medicine, Soonchunhyang University Seoul Hospital, 59 Daesagwan-ro, Yongsan-gu, Seoul, 04401 Korea; 2 Hyonam Kidney Laboratory, Soonchunhyang University Seoul Hospital, 59 Daesagwan-ro, Yongsan-gu, Seoul, 04401 Korea; 3 Department of Biostatistics, Soonchunhyang University Seoul Hospital, 59 Daesagwan-ro, Yongsan-gu, Seoul, 04401 Korea

**Keywords:** Diseases, Genetics, Nephrology, Risk factors

## Abstract

The *Aldehyde dehydrogenase 2* (*ALDH2*) rs671 polymorphism, a common variant that impairs aldehyde detoxification, has been linked to cardiovascular disease, but its role in chronic kidney disease (CKD) remains unclear. This study examined the association between the *ALDH2* rs671 polymorphism and incident CKD in a population-based cohort, and whether alcohol consumption modifies this relationship. We analyzed 5,369 Korean adults aged 40–69 years without CKD at baseline from the community-based Korean Genome and Epidemiology Stud**y**, followed biennially for up to 18 years. The main exposures were *ALDH2* genotype (GG vs. GA/AA) and categorized alcohol consumption (none, low, moderate, high). Incident CKD was defined as an estimated glomerular filtration rate (eGFR) < 60 mL/min/1.73 m² or new-onset proteinuria (≥ 1 + on dipstick). Cox proportional hazards models estimated adjusted hazard ratios (HRs). During a mean 11.7-year follow-up, 1,396 participants (26.0%) developed CKD. CKD risk did not differ significantly between genotypes, and alcohol intake was not associated with CKD incidence. These associations were consistent across genotype or sex. Overall, *ALDH2* rs671 and alcohol intake showed limited relevance to CKD onset, suggesting that *ALDH2*-related biological effects may be more pertinent to disease progression rather than initiation in the general population.

**Supplementary Information:**

The online version contains supplementary material available at 10.1038/s41598-026-43186-4.

## Introduction

*Aldehyde dehydrogenase 2 (ALDH2)* is a mitochondrial enzyme essential for the detoxification of reactive aldehydes, particularly acetaldehyde, a byproduct of ethanol metabolism^1^. Because of their high reactivity with DNA and proteins, cytotoxic aldehydes can impair cellular processes and promote genetic instability^2,3^. The *ALDH2* rs671 polymorphism (Glu504Lys) impairs ALDH2 enzymatic activity, reducing the clearance of these aldehydes generated from alcohol metabolism^4^. This variant is common, affecting approximately 8% of the global population and 35–45% of East Asians^5^. ALDH2 has a role not only in alcohol metabolism but also in antioxidant defense mechanism. The rs671 variant has been associated with several cardiovascular diseases (CVDs), including macrovascular events in diabetes mellitus, diabetic retinopathy, hypertension, coronary artery spasm, and acute myocardial infarction, particularly in East Asian populations^6–10^.

Chronic kidney disease (CKD) affects 9–10% of the global population and represents a significant public health burden. Experimental studies have suggested a role for ALDH2 in kidney disease, particularly in the context of disease progression. Mice with reduced ALDH2 activity exhibit increased susceptibility to renal fibrosis, while enhancement of ALDH2 expression in proximal tubular cells attenuates transforming growth factor-β (TGF-β)–mediated epithelial dedifferentiation and fibrotic remodeling^11^. Experimental studies indicate that ALDH2 deficiency does not induce spontaneous kidney disease under baseline conditions^12^, but rather exacerbates renal fibrosis and injury following established damage, suggesting a role in disease progression rather than initiation. Consistent with these experimental findings, several genome-wide association studies (GWAS) reported associations between *ALDH2* and continuous indicators of renal function, such as blood urea nitrogen, serum creatinine, estimated glomerular filtration rate, and proteinuria^13–15^. However, the relationship between the *ALDH2* variant and incident CKD in human cohorts has yet to be clearly established.

Research examining the impact of alcohol consumption on kidney function has yielded inconsistent findings^16^. According to the National Kidney Foundation, heavy alcohol intake may double the risk of kidney disease^17^. Additionally, *ALDH2* variants have been linked to susceptibility to essential hypertension, a major risk factor for CKD^18^. In contrast, some studies have reported that moderate alcohol intake is associated with a lower CKD risk compared with abstinence^19^. The *ALDH2* variant may influence drinking behavior and limit the detoxification of harmful alcohol metabolites^20^, which together may affect an individual’s vulnerability to alcohol-related kidney injury.

Therefore, we aimed to examine whether the *ALDH2* rs671 polymorphism is associated with incident CKD in a general population, and to determine whether impaired aldehyde metabolism and alcohol exposure contribute to the initiation of CKD. Furthermore, we evaluated whether alcohol consumption modifies this association, considering the potential interaction between impaired aldehyde metabolism and alcohol exposure. To address these aims, we analyzed data from a large, nationwide, prospective cohort of middle-aged Korean adults.

## Methods

### Study participants

The Korean Genome and Epidemiology Study (KoGES) Ansan and Ansung is a prospective, community-based cohort that includes health surveys, physical examinations, laboratory tests, and genome-wide association studies. Cohort data are available through the Korea Disease Control and Prevention Agency. Detailed cohort methodology has been previously described^21^. KoGES Ansan and Ansung comprises 9,333 participants aged 40–69 years, all of Korean ethnicity and residing in either Ansan or Ansung in Gyeonggi Province, South Korea. Genomic data were available for 5,617 individuals. After excluding 124 participants with prevalent CKD at baseline, 5,493 participants were included in the final analysis. The cohort was recruited between 2001 and 2002, with biennial follow-up assessments. Data from the eighth follow-up, completed in 2018, were used for this study.

All participants provided written informed consent. This study adhered to the ethical principles of the Declaration of Helsinki and was approved by the Ethics Committee of the Korean Health and Genomic Study at the Korea National Institute of Health and the Institutional Review Board of Soonchunhyang University Hospital (No. 2023-03−004).

### Definition of variables

Participants completed health surveys and underwent clinical assessments at designated centers using standardized protocols implemented by trained interviewers and examiners. The surveys collected detailed information on lifestyle behaviors, medical and surgical history, family history, occupation, education, smoking status, alcohol intake, physical activity, psychosocial factors, and diet. Data were collected biennially.

Trained staff measured anthropometric indices and blood pressure using standardized procedures. Height, weight, and waist circumference (WC) were recorded using automated devices. Body mass index (BMI) was calculated as weight divided by height squared (kg/m²). Blood pressure was measured using a mercury sphygmomanometer following ≥ 5 min of seated rest; repeated measurements were taken until the final two readings differed by < 5 mmHg.

Participants were classified as having hypertension (HTN) if they met any of the following: systolic blood pressure (SBP) ≥ 140 mmHg, diastolic blood pressure (DBP) ≥ 90 mmHg, physician-diagnosed hypertension, or current use of antihypertensive medications. Diabetes mellitus was defined as having any of the following: fasting glucose ≥ 126 mg/dL after ≥ 8 h of fasting, postprandial glucose ≥ 200 mg/dL, hemoglobin A1c (HbA1c) ≥ 6.5%, or current use of oral hypoglycemic agents or insulin. Dyslipidemia and cardiovascular disease were determined by self-report or medical diagnosis of myocardial infarction, heart failure, coronary artery disease, or stroke.

Fasting blood samples were analyzed for creatinine, albumin, glucose, total cholesterol, triglycerides, high-density lipoprotein (HDL) cholesterol, and high-sensitivity C-reactive protein. Low-density lipoprotein (LDL) cholesterol was calculated using a standard formula. Serum creatinine was measured by the Jaffe method and calibrated to ensure comparability with isotope dilution mass spectrometry (IDMS). The estimated glomerular filtration rate (eGFR) was calculated using the CKD Epidemiology Collaboration (CKD-EPI) equation. HbA1c was measured using standardized laboratory methods. Proteinuria was assessed using dipstick analysis of morning urine samples and defined as ≥ 1+.

Socioeconomic variables included income and educational attainment. Income level was classified based on average monthly household income into three categories: low (< 1.5 million Korean won [KRW]), intermediate (1.5–2.5 million KRW), and high (≥ 2.5 million KRW). Education was categorized as elementary school or less, middle school or less, and high school or higher. Physical activity was assessed via questionnaire and categorized according to the World Health Organization guideline of ≥ 150 min per week of moderate-to-vigorous physical activity (MVPA)^22^. Participants were grouped into two categories based on total weekly activity (including vigorous exercise and walking): <150 min per week and ≥ 150 min per week.

### Study outcomes

The primary endpoint was incident CKD, defined as a composite of decreased eGFR < 60 mL/min/1.73m^2^ or new-onset proteinuria during the follow-up period. Participants who died during follow-up were censored at the time of death. Alcohol consumption was analyzed as an explanatory variable, and subgroup analyses assessed its potential modifying effect on CKD incidence across *ALDH2* genotypes.

### Alcohol consumption

Alcohol intake data were obtained via standardized interviews. Participants were asked whether they consumed at least one alcoholic drink per month. Daily alcohol consumption was estimated based on reported drinking frequency, average volume per occasion, and the alcohol content of a standard drink. Participants were categorized into four groups by daily alcohol consumption: non-drinkers, low (< 5 g/day), moderate (5–30 g/day), and high (≥ 30 g/day) drinkers. This categorization was based on previous Korean population-based studies, which identified 5 g/day as a threshold for metabolically relevant alcohol exposure^23,24^.

### Genotyping and classification of ALDH2 variants

Genotyping data for the *ALDH2* rs671 polymorphism were extracted from the KoGES cohort using the Korea Biobank Array (KBA), a custom-designed platform optimized for the Korean population^25^. The KBA includes > 833,000 markers, including > 247,000 rare or functional variants identified through sequencing of > 2,500 Korean individuals^26^. Standard quality control measures were applied. Samples were excluded if they had a call rate < 97%, excessive heterozygosity or singletons, sex discrepancies, or cryptic first-degree relationships. SNPs with Hardy–Weinberg equilibrium p-values < 1 × 10⁻⁶ or call rates < 95% were also excluded. After quality control, data were phased using Eagle v2.3 and imputed using IMPUTE4 with the 1000 Genomes Project Phase 3 and a Korean reference panel^26^. Participants were classified by genotype as GG or GA/AA.

### Statistical analysis

Continuous variables are presented as medians with interquartile ranges (IQRs), and categorical variables as counts and percentages. Normally distributed continuous variables were compared using the t-test, while non-normally distributed data were analyzed using the Mann–Whitney U test. Categorical variables were compared using the Chi-squared or Fisher’s exact test. Kaplan–Meier curves with log-rank tests were used to compare cumulative CKD incidence across *ALDH2* genotypes. Participants lost to follow-up or deceased were censored at the time of last contact.

Cox proportional hazards models were used to estimate hazard ratios (HRs) and 95% confidence intervals (CIs) for incident CKD. Unadjusted models included only the primary exposure, whereas multivariable-adjusted models included alcohol consumption, age, sex, body mass index, diabetes mellitus, hypertension, dyslipidemia, smoking status, physical activity, income, educational status, hemoglobin level, and baseline estimated glomerular filtration rate. The proportional hazards assumption was assessed using Schoenfeld residuals.

To evaluate potential effect modification, stratified analyses were conducted by *ALDH2* genotype (GG vs. GA/AA) and sex (male vs. female). Multivariable-adjusted Cox models were fitted within each subgroup using the same set of covariates. In addition, a multiplicative interaction term between *ALDH2* genotype and alcohol consumption was included in the Cox model to formally test for interaction. A two-sided p-value < 0.05 was considered statistically significant. All analyses were performed using R version 4.3.1.

## Results

### Baseline characteristics

A total of 5,369 participants from the KoGES Ansan and Ansung cohorts were included after excluding 124 individuals with CKD at baseline. The median age was 49 years (IQR, 44–59), and 2,580 participants (48.0%) were male. The mean follow-up duration from 2001 to 2018 was 11.67 ± 4.05 years. Based on *ALDH2* genotyping, 3,818 participants (71.1%) had the GG genotype, while 1,411 (26.8%) and 140 (2.6%) had GA or AA genotypes, respectively. Table 1 presents baseline characteristics by genotype. No clinically meaningful differences were observed in blood pressure, BMI, or smoking status between the GG group and the GA or AA group. The prevalence of comorbid conditions—hypertension, diabetes mellitus, and dyslipidemia—was also similar across genotypes. Socioeconomic indicators, including income and education level, showed no significant difference between groups.

**Table 1:** Baseline characteristics by *ALDH2* genotype.

	GG (*n* = 3,818)	GA + AA (*n* = 1,551)	Total (*n* = 5,369)	*p* value
Age, years, median [IQR]	49 (44–58)	50 (44–59)	49 (44–59)	0.144
Males, n (%)	1,827 (47.85)	753 (48.55)	2,580 (48.05)	0.665
SBP, mmHg, median [IQR]	119 (109–131)	117 (108–130)	119 (108–131)	0.057
DBP, mmHg, median [IQR]	80 (72–88)	79 (71–87)	80 (72–88)	0.027
BMI, kg/m^2^, median [IQR]	24.6 (22.7–26.6)	24.4 (22.4–26.4)	24.5 (22.6–26.5)	0.023
Smoking				0.574
Never, n (%)	2,246 (59.5)	907 (59.3)	3,153 (59.5)	
Former, n (%)	616 (16.3)	257 (16.8)	873 (16.5)	
Current, n (%)	911 (24.2)	365 (23.9)	1276 (24.1)	
Alcohol intake				< 0.001
Never, n (%)	1,365 (39.5%)	1,040 (72.4%)		
Low, n (%)	686 (19.8%)	210 (14.6%)		
Moderate, n (%)	866 (25.0%)	145 (10.1%)		
High, n (%)	541 (15.6%)	41 (2.9%)		
Total alcohol consumption, g/day, median [IQR]	11.6 (3.3–28.9)	4.2 (1.5–11.8)		< 0.001
Medical history				
HTN, n (%)	574 (15.0)	204 (13.2)	778 (14.5)	0.084
DM, n (%)	251 (6.6)	78 (5.0)	329 (6.1)	0.038
Dyslipidemia, n (%)	104 (2.7)	39 (2.5)	143 (2.7)	0.736
MVPA, n (%)	2,235 (60.3)	831 (60.9)	3,066 (61.5)	0.902
Income				0.912
Low, n (%)	1,819 (48.4)	739 (48.4)	2,558 (48.4)	
Intermediate, n (%)	1,231 (32.8)	493 (32.3)	1,724 (32.6)	
High, n (%)	707 (18.8)	294 (19.3)	1,001 (19.0)	
Education				0.9
Low, n (%)	1,149 (30.3)	469 (30.4)	1,618 (30.3)	
Middle, n (%)	870 (22.9)	353 (22.9)	1,223 (22.9)	
High, n (%)	1,773 (46.8)	721 (46.7)	2,494 (46.8)	
Hb, g/dL, median [IQR]	13.5 (12.5–14.8)	13.6 (12.5–14.8)	13.6 (12.5–14.8)	0.295
Alb, g/dL, median [IQR]	4.5 (4.3–4.7)	4.5 (4.3–4.7)	4.5 (4.3–4.7)	0.084
T.chol, mg/dL, median [IQR]	197 (174–223)	195 (173–219)	196 (174–221)	0.233
HDL, mg/dL, median [IQR]	48 (42–56)	46 (40–54)	48 (41–56)	< 0.001
LDL, mg/dL, median [IQR]	117.8 (96.2–140.2)	119.6 (99.4–142)	118.2 (97.2–140.8)	0.006
TG, mg/dL, median [IQR]	125 (87–187)	121 (86–171.5)	124 (87–182)	0.003
HbA1c, %, median [IQR]	5.6 (5.3–5.9)	5.6 (5.3–5.9)	5.6 (5.3–5.9)	0.680
FBS, mg/dL, median [IQR]	88 (83–95)	87 (82–93)	88 (83–95)	< 0.001
BUN, mg/dL, median [IQR]	13.4 (11.2–16.0)	13.7 (11.5–16.3)	13.5 (11.3–16)	0.004
Cr, mg/dL, median [IQR]	0.8 (0.7–1)	0.8 (0.7–1)	0.8 (0.7–1)	0.492
eGFR, median [IQR)	95.5 (82.4–105.2)	94.48 (81.4–105.0)	95.1 (81.8–105.0)	0.306

*ALDH2*, aldehyde dehydrogenase 2; SBP, systolic blood pressure; DBP, diastolic blood pressure; BMI, body mass index; HTN, hypertension; DM, diabetes mellitus; MVPA, moderate-to-vigorous physical activity; Hb, hemoglobin; Alb, albumin; T.chol, total cholesterol; HDL, high-density lipoprotein; LDL, low-density lipoprotein; TG, triglycerides; HBa1c, hemoglobin A1c; FBS, fasting blood sugar; BUN, blood urea nitrogen; Cr, creatinine; eGFR, estimated glomerular filtration rate; IQR, interquartile range.

Alcohol intake, categorized as low (< 5 g/day), moderate (5–30 g/day), or high (≥ 30 g/day); Income level, categorized as low (< 1.5 million Korean won/month), intermediate (1.5–2.5 million won/month), or high (≥ 2.5 million won/month); Educational level, classified as low (elementary school or less), middle (up to middle school), or high (high school or higher).

### Alcohol consumption according to *ALDH2* genotype

Among participants with the GG genotype, 15.4% were high alcohol consumers, 25.0% moderate, and 19.8% low (Table 1). In contrast, among those with the GA or AA genotypes, 2.6% were high, 9.3% moderate, and 15.5% low alcohol consumers, while 67.6% were non-drinkers. Thus, participants with the GG genotype consumed significantly more alcohol than those with the GA or AA genotype (*p* < 0.001).

### Association of *ALDH2* genotype and alcohol consumption with incident CKD

**Fig. 1:**
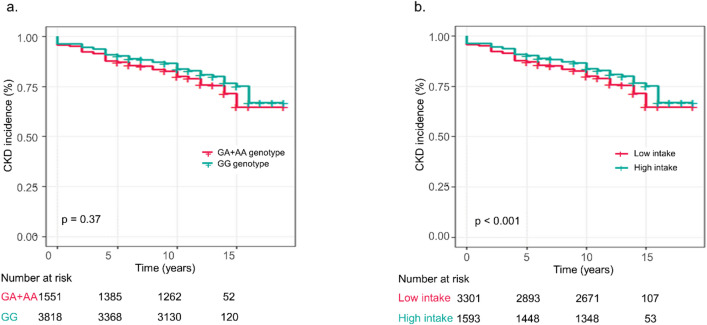
Kaplan–Meier curves for incident CKD according to *ALDH2* genotype and alcohol consumption. **(A)** Cumulative CKD-free survival according to *ALDH2* genotype (GG vs. GA + AA; log-rank *p* = 0.37). **(B)** Cumulative CKD-free survival according to alcohol consumption (≥ 5 g/day vs. < 5 g/day; log-rank *p* < 0.001). *ALDH2*, aldehyde dehydrogenase 2; *CKD*, chronic kidney disease.

During the mean 11.67-year follow-up, CKD developed in 1,396 participants (26.0%). Of these, 1,007 cases (26.4%) occurred in the GG group (*n* = 3,818), and 389 cases (25.1%) in the GA or AA group (*n* = 1,551). There was no significant difference in CKD incidence between *ALDH2* genotypes (GG vs. GA or AA; log-rank *p* = 0.37; Fig. 1A), indicating that the *ALDH2* genotype itself was not associated with the risk of CKD in the overall cohort.

In the overall cohort, participants with moderate-to-high alcohol consumption (≥ 5 g/day) showed a lower incidence of CKD than non- or low drinkers (< 5 g/day) in the unadjusted analysis (log-rank *p* < 0.001; Fig. 1B). However, this inverse association disappeared after multivariable adjustment in the Cox model (Table 2A, “Alcohol consumption” row), suggesting confounding by factors such as age, sex, and comorbidities. No statistically significant interaction between *ALDH2* genotype and alcohol consumption was observed when a multiplicative interaction term was included in the Cox model (p for interaction = 0.804). Detailed coefficients for all covariates included in the multivariable model are provided in Supplementary Table S1.

**Table 2:** Cox proportional hazards models for incident CKD.

	Unadjusted		Adjusted*	
	HR (95% CI)	*p* value	HR (95% CI)	*p* value
(A) Overall cohort				
*ALDH2* genotype, GG/(GA + AA)	**1.03** (0.92–1.17)	0.584	**1.08** (0.94–1.25)	0.295
Alcohol consumption, (Moderate + High/None + Low)	**0.78 (0.69–0.89)**	**< 0.001**	**1.10** (0.78–1.55)	0.558
*ALDH2* genotype * alcohol consumption			0.66 (0.36–1.21)	0.178
(B) Stratified by genotype				
**GG genotype (*n*** **= 3818)**				
Alcohol consumption, (high ≥ 5 g/day/Low < 5 g/day)	0.75 (0.65–0.86)	< 0.001	0.92 (0.74–1.15)	0.473
**GA + AA genotype (*n*** **= 1551)**				
Alcohol consumption, (high ≥ 5 g/day/Low < 5 g/day)	0.87 (0.63–1.21)	0.418	1.22 (0.84–1.78)	0.289

*ALDH2*, aldehyde dehydrogenase 2; CKD, chronic kidney disease; HR, hazard ratio; CI, confidence interval.

* Adjusted for age, sex, BMI, DM, HTN, hyperlipidemia, smoking status, MVPA, Hb, income, education and eGFR.

**Fig. 2:**
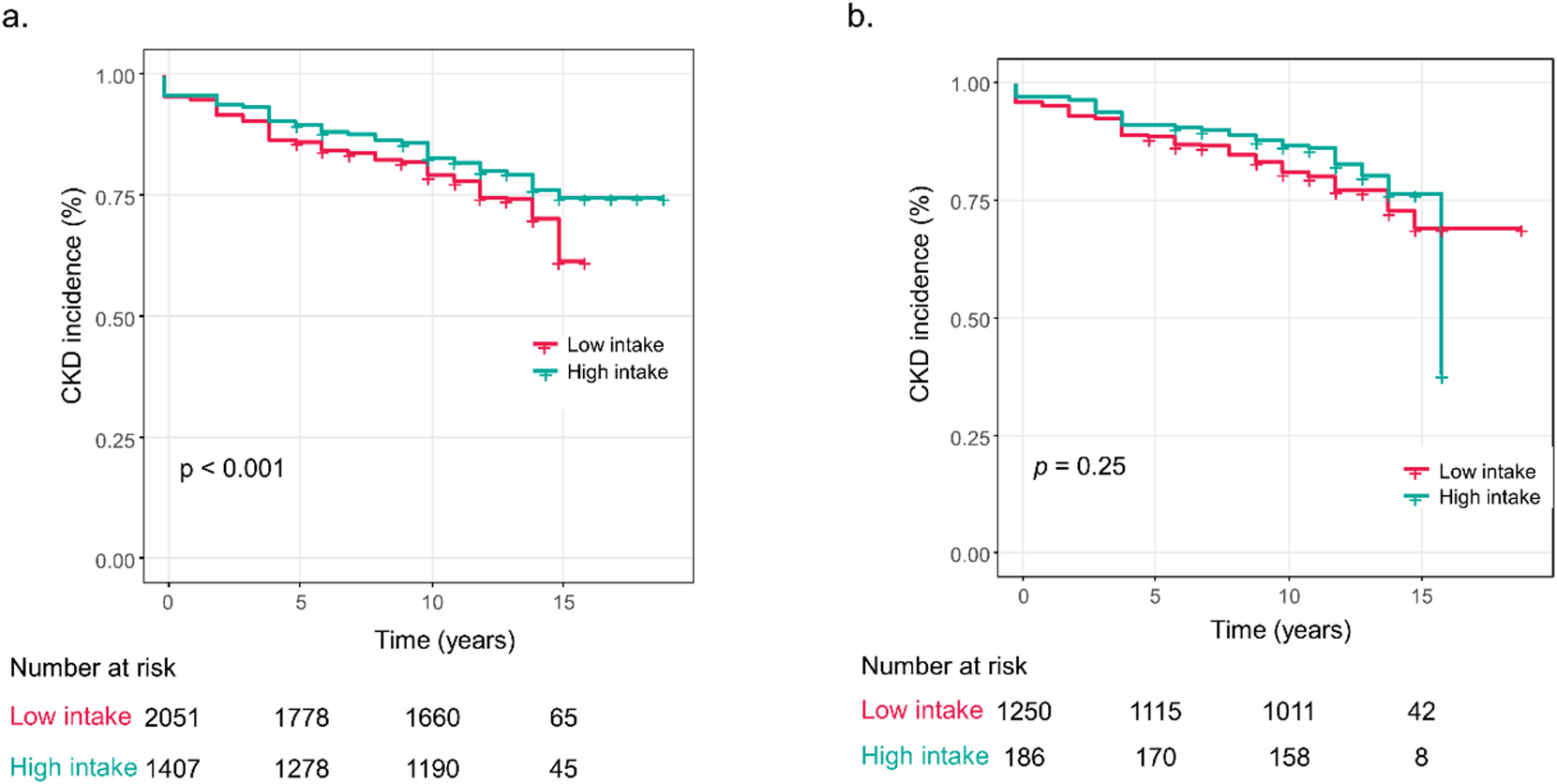
Stratified analysis of alcohol consumption and incident CKD according to *ALDH2* genotype. **(A)** Kaplan–Meier curves comparing CKD-free survival between alcohol groups (≥ 5 g/day vs. < 5 g/day) in GG genotypes. **(B)** Kaplan–Meier curves comparing CKD-free survival between alcohol groups (≥ 5 g/day vs. < 5 g/day) in GA/AA genotypes. *ALDH2*,* aldehyde dehydrogenase 2; CKD*,* chronic kidney disease; HR*,* hazard ratio; CI*,* confidence interval.*

When the analysis was stratified by genotype (Table 2B and Fig. 2), a lower CKD risk among moderate-to-high drinkers was observed only in the GG group in the unadjusted analysis (HR, 0.75 [95% CI 0.65–0.86]; *p* < 0.001), but the association became non-significant after adjustment (HR 0.92 [0.74–1.15]; *p* = 0.47). In the GA or AA group, alcohol intake was not associated with CKD risk in either the unadjusted or multivariable-adjusted analyses (adjusted HR 1.22, 95% CI 0.84–1.78; *p* = 0.29). Adjusted hazard ratios and 95% CIs for each subgroup are summarized in Table 2.

In sex-stratified analyses (Supplementary Tables S4 and S5), the direction of association between alcohol consumption and incident CKD was similar in men and women after adjustment. Among men with the GG genotype, moderate-to-high alcohol intake was not significantly associated with CKD risk in either univariate (HR 0.82 [0.64–1.05], *p* = 0.11) or multivariate analyses (HR 0.93 [0.72–1.20], *p* = 0.57). Among women with the GG genotype, the unadjusted association (HR, 0.57 [0.38–0.86]; *p* = 0.007) also lost significance after adjustment (HR, 0.90 [0.59–1.38]; *p* = 0.64**).** Taken together, there was no clear evidence that sex modified the association between alcohol consumption and CKD risk.

### Sensitivity analysis

To address potential heterogeneity in the CKD definition, we performed sensitivity analyses using eGFR decline and albuminuria as separate outcomes. Neither ALDH2 genotype nor alcohol consumption was significantly associated with incident eGFR decline or albuminuria after multivariable adjustment, and no significant interaction between genotype and alcohol consumption was observed for either outcome (Supplementary Tables S2 and S3).

## Discussion

In this large, prospective Korean cohort, *ALDH2* rs671 was not associated with incident CKD, and we found no evidence that alcohol consumption modified this association. These findings remained consistent across multivariable models and subgroup analyses. Although prior experimental and genetic studies have implicated *ALDH2* in renal injury, our results suggest that *ALDH2* rs671 is unlikely to be a major independent determinant of CKD onset at the population level in this general population cohort. GWAS have linked *ALDH2*-related loci to kidney function traits such as serum creatinine and eGFR^13,15^. However, these associations with biomarkers do not necessarily translate into clinically overt CKD. Our findings extend this literature by providing prospective human evidence that genetic associations with kidney biomarkers may be insufficient to influence the development of CKD at the population level. This null association was also observed in sensitivity analyses that examined incident eGFR decline and incident albuminuria separately (Supplementary Tables S2 and S3), which reduces concern that our primary findings were driven solely by heterogeneity in the composite CKD definition.

A plausible explanation is that ALDH2 deficiency may be more relevant to CKD progression than to disease initiation. Experimental studies, including a model published in *Kidney International*^11^, have demonstrated that ALDH2 suppression exacerbates oxidative stress and TGF-β–mediated fibrotic pathways in the kidney, leading to aggravated renal fibrosis under conditions of established kidney injury, particularly toxin-induced tubular damage and TGF-β–driven profibrotic signaling. Importantly, these experimental settings reflect pre-existing renal injury, rather than the early stages of kidney dysfunction captured in population-based cohort studies. Therefore, our null association with incident CKD does not necessarily contradict mechanistic evidence; rather, it suggests that ALDH2-related pathways may become clinically consequential after injury has occurred, potentially influencing fibrosis, functional decline trajectories, or progression to advanced CKD. Future studies focusing on longitudinal eGFR slope, albuminuria trajectories, and fibrosis-related biomarkers would be better suited to test this hypothesis.

Prior observational studies frequently reported an inverse or J-shaped relationship between alcohol intake and CKD risk^27–29^. In our cohort, a similar inverse association was observed among individuals with the GG genotype, but it was substantially attenuated after accounting for *ALDH2* genotype and other confounders. Taken together, these results suggest that previously reported “protective” associations may partly reflect confounding and selection effects, and that genetic susceptibility to alcohol metabolism does not translate into differential CKD incidence in this setting.

Several additional factors may also contribute to the observed null findings. CKD incidence in the general population is shaped by multiple metabolic, vascular, and inflammatory pathways, which may overshadow modest effects of a single variant. Moreover, humans may compensate for reduced ALDH2 activity through other aldehyde-detoxifying systems, including glutathione-dependent pathways^30^. Finally, because most participants were light-to-moderate drinkers, cumulative acetaldehyde exposure may have been insufficient to trigger clinically detectable CKD onset, even among genetically susceptible individuals.

We defined low alcohol exposure using a 5 g/day threshold, consistent with prior KoGES-based studies^23,24^. Previous studies have identified 5 g/day as a metabolically significant increment for various chronic conditions in the Korean population^31^. Furthermore, considering that individuals with the *ALDH2* rs671 variant exhibit a marked increase in blood acetaldehyde levels even after minimal alcohol intake, this more conservative threshold is clinically more relevant for capturing the potential toxic effects of alcohol in a genetically susceptible East Asian population.

We also found no sex-specific associations between alcohol use and the incidence of CKD, despite prior evidence suggesting greater vulnerability to alcohol-related toxicity in women and a possible protective effect in men. The absence of sex-specific differences in our study may reflect cultural or behavioral patterns in alcohol consumption or cohort-specific characteristics that offset biological susceptibility.

Key strengths include the prospective design with long follow-up, a large ethnically homogeneous cohort with standardized assessment of kidney outcomes, and extensive adjustment for established CKD risk factors. Integrating *ALDH2* genotype with alcohol exposure provides population-based evidence that genetic variation in alcohol metabolism has limited influence on CKD onset in this setting.

### Study limitations

Several limitations should be acknowledged. First, alcohol consumption was self-reported and therefore subject to under-reporting and misclassification, which may have attenuated true associations. Second, CKD was defined using eGFR and dipstick proteinuria rather than quantitative albuminuria or repeated confirmatory measurements; thus, our outcome may have included transient eGFR reductions or temporary proteinuria, introducing heterogeneity and potentially diluting modest genetic effects. Third, although extensive covariates were adjusted for, residual confounding by unmeasured biological, genetic, or environmental factors cannot be fully excluded. Fourth, because we focused on incident CKD and did not evaluate post-onset trajectories, we may have underestimated any potential role of ALDH2 in CKD progression or fibrotic remodeling. Finally, the generalizability of our findings may be limited to East Asian populations, in whom *ALDH2* variants are common, and to drinking patterns characteristic of Korean society.

Despite these limitations, the large sample size, long-term prospective follow-up, and standardized assessment of kidney outcomes support the robustness of our conclusions.

## Conclusion

In this prospective cohort of Korean adults, *ALDH2* rs671 genotype was not associated with incident CKD, nor was this association modified by alcohol consumption or sex. These findings suggest a limited role of *ALDH2*-mediated alcohol metabolism in CKD initiation, while potential effects on disease progression warrant further investigation.

## Supplementary Information

Below is the link to the electronic supplementary material.

Supplementary Material 1

## Data Availability

The data that support the findings of this study are available from the Korean Genome and Epidemiology Study (KoGES), Korea Disease Control and Prevention Agency. Restrictions apply to the availability of these data, which were used under license for the current study and are not publicly available. Data access can be obtained upon reasonable request and with permission of the data provider.

## References

[1] Zakhari, S. Overview: how is alcohol metabolized by the body? *Alcohol Res. Health*. **29** (4), 245–254 (2006). 17718403 PMC6527027

[2] Chen, C. H., Sun, L. & Mochly-Rosen, D. Mitochondrial aldehyde dehydrogenase and cardiac diseases. *Cardiovasc. Res. Oct.* **1** (1), 51–57. 10.1093/cvr/cvq192 (2010). 10.1093/cvr/cvq192 PMC2936126 20558439

[3] Jin, S. et al. ALDH2(E487K) mutation increases protein turnover and promotes murine hepatocarcinogenesis. *Proc. Natl. Acad. Sci. U S Jul*. **21** (29), 9088–9093. 10.1073/pnas.1510757112 (2015). 10.1073/pnas.1510757112 PMC4517197 26150517

[4] Xu, H., Zhang, Y. & Ren, J. ALDH2 and stroke: a systematic review of the evidence. *Adv. Exp. Med. Biol.* **1193**, 195–210. 10.1007/978-981-13-6260-6_11 (2019). 31368105 10.1007/978-981-13-6260-6_11

[5] Enomoto, N., Takase, S., Yasuhara, M. & Takada, A. Acetaldehyde metabolism in different aldehyde dehydrogenase-2 genotypes. *Alcohol Clin. Exp. Res. Feb*. **15** (1), 141–144. 10.1111/j.1530-0277.1991.tb00532.x (1991). 10.1111/j.1530-0277.1991.tb00532.x 2024727

[6] Idewaki, Y. et al. Association of genetically determined aldehyde dehydrogenase 2 activity with diabetic complications in relation to alcohol consumption in Japanese patients with type 2 diabetes mellitus: the Fukuoka Diabetes Registry. *PLoS One*. **10** (11), e0143288. 10.1371/journal.pone.0143288 (2015). 26599441 10.1371/journal.pone.0143288 PMC4658066

[7] Morita, K. et al. Association between aldehyde dehydrogenase 2 polymorphisms and the incidence of diabetic retinopathy among Japanese subjects with type 2 diabetes mellitus. *Cardiovasc. Diabetol. Sep. 13*. **12**, 132. 10.1186/1475-2840-12-132 (2013). 10.1186/1475-2840-12-132 PMC3847457 24028448

[8] Wu, H., Huang, Q., Yu, Z. & Zhong, Z. Association of ALDH2 rs671 and MTHFR rs1801133 polymorphisms with hypertension among Hakka people in Southern China. *BMC Cardiovasc. Disord Mar.* **27** (1), 128. 10.1186/s12872-022-02577-x (2022). 10.1186/s12872-022-02577-x PMC8962465 35346052

[9] Mizuno, Y. et al. Variant aldehyde dehydrogenase 2 (ALDH2*2) is a risk factor for coronary spasm and ST-segment elevation myocardial infarction. *J Am Heart Assoc.* **5**(5), e003247. (2016). 10.1161/JAHA.116.003247 10.1161/JAHA.116.003247 PMC4889196 27153870

[10] Xia, C. L. et al. ALDH2 rs671 polymorphism and the risk of heart failure with preserved ejection fraction (HFpEF) in patients with cardiovascular diseases. *J. Hum. Hypertens. Jan*. **34** (1), 16–23. 10.1038/s41371-019-0182-2 (2020). 10.1038/s41371-019-0182-2 30846829

[11] Chiu, I. J. et al. Suppression of aldehyde dehydrogenase 2 in kidney proximal tubules contributes to kidney fibrosis through Transforming Growth Factor-β signaling. *Kidney Int. Jan*. **107** (1), 84–98. 10.1016/j.kint.2024.09.010 (2025). 10.1016/j.kint.2024.09.010 39393529

[12] Li, S. Y. et al. Aldehyde dehydrogenase 2 preserves kidney function by countering acrolein-induced metabolic and mitochondrial dysfunction. *JCI Insight Oct.* **8** (19), e179871. 10.1172/jci.insight.179871 (2024). 10.1172/jci.insight.179871 PMC11466184 39226171

[13] Okada, Y. et al. Meta-analysis identifies multiple loci associated with kidney function-related traits in East Asian populations. *Nat. Genet. Jul*. **15** (8), 904–909. 10.1038/ng.2352 (2012). 10.1038/ng.2352 PMC4737645 22797727

[14] Ledo, N. et al. Functional genomic annotation of genetic risk loci highlights inflammation and epithelial biology networks in CKD. *J. Am. Soc. Nephrol. Mar.* **26** (3), 692–714. 10.1681/ASN.2014010028 (2015). 10.1681/ASN.2014010028 PMC4341476 25231882

[15] Lee, J. et al. Genome-wide association analysis identifies multiple loci associated with kidney disease-related traits in Korean populations. *PLoS One*. **13** (3), e0194044. 10.1371/journal.pone.0194044 (2018). 29558500 10.1371/journal.pone.0194044 PMC5860731

[16] Fan, Z., Yun, J., Yu, S. & Y, Q. Alcohol consumption can be a double-edged sword for chronic kidney disease patients. *Med. Sci. Monit.* **25**, 7059–7072. 10.12659/MSM.916121 (2019). 31538630 10.12659/MSM.916121 PMC6767945

[17] Foundation, N. K. Alcohol and your kidneys. National Kidney Foundation. Accessed April 19, (2025). https://www.kidney.org/kidney-topics/alcohol-and-your-kidneys

[18] Wu, Y. et al. Positive association between ALDH2 rs671 polymorphism and essential hypertension: A case-control study and meta-analysis. *PLoS One*. **12** (5), e0177023. 10.1371/journal.pone.0177023 (2017). 28472173 10.1371/journal.pone.0177023 PMC5417637

[19] Lai, Y. J. et al. Alcohol consumption and risk of chronic kidney disease: a nationwide observational cohort study. *Nutrients Sep.* **6** (9), 2121. 10.3390/nu11092121 (2019). 10.3390/nu11092121 PMC6769971 31489891

[20] Lin, C. L. et al. The aldehyde dehydrogenase ALDH2*2 allele, associated with alcohol drinking behavior, dates back to prehistoric times. *Biomolecules Sep.* **17** (9), 1376. 10.3390/biom11091376 (2021). 10.3390/biom11091376 PMC8465343 34572589

[21] Kim, Y., Han, B. G. & Group, K. Cohort profile: the Korean Genome and Epidemiology Study (KoGES) Consortium. *Int. J. Epidemiol. Apr*. **1** (2), e20. 10.1093/ije/dyv316 (2017). 10.1093/ije/dyv316 PMC5837648 27085081

[22] World Health Organization. Global recommendations on physical activity for health. WHO/9789241599979. (2010). https://iris.who.int/bitstream/handle/10665/44399/9789241599979_eng.pdf 26180873

[23] Baik, I. & Shin, C. Prospective study of alcohol consumption and metabolic syndrome. *Am. J. Cln Nutr.* **87** (5), 1455–1463. 10.1093/ajcn/87.5.1455 (2008). 10.1093/ajcn/87.5.1455 18469271

[24] Yoo, M. et al. National distribution of rs671 (ALDH2) genotypes and the effect of alcohol on chronic disease in each genotype. *Public. Health Wkly. Rep.* **14** (29), 2095–2110 (2021).

[25] Moon, S., Kim, Y. J. & Han, S. et al. The Korea Biobank Array: design and identification of coding variants associated with blood biochemical traits. *Sci. Rep. Feb*. **4** (1), 1382. 10.1038/s41598-018-37832-9 (2019). 10.1038/s41598-018-37832-9 PMC6361960 30718733

[26] Nam, K., Kim, J. & Lee, S. Genome-wide study on 72,298 individuals in Korean biobank data for 76 traits. *Cell. Genom Oct.* **12** (10), 100189. 10.1016/j.xgen.2022.100189 (2022). 10.1016/j.xgen.2022.100189 PMC9903843 36777999

[27] Yamamoto, R. et al. A Dose-Dependent Association between Alcohol Consumption and Incidence of Proteinuria and Low Glomerular Filtration Rate: A Systematic Review and Meta-Analysis of Cohort Studies. *Nutrients. * **15**(7). 10.3390/nu15071592 (2023). 10.3390/nu15071592 PMC10097279 37049433

[28] Koning, S. H. et al. Alcohol consumption is inversely associated with the risk of developing chronic kidney disease. *Kidney Int. May*. **87** (5), 1009–1016. 10.1038/ki.2014.414 (2015). 10.1038/ki.2014.414 25587707

[29] White, S. L. et al. Alcohol consumption and 5-year onset of chronic kidney disease: the AusDiab study. *Nephrol. Dial Transpl. Aug*. **24** (8), 2464–2472. 10.1093/ndt/gfp114 (2009). 10.1093/ndt/gfp114 19307230

[30] Gao, J., Hao, Y., Piao, X. & Gu, X. Aldehyde Dehydrogenase 2 as a Therapeutic Target in Oxidative Stress-Related Diseases: Post-Translational Modifications Deserve More Attention. *Int. J. Mol. Sci. ***23** (5). 10.3390/ijms23052682 (2022). 10.3390/ijms23052682 PMC8910853 35269824

[31] Yoo, M. G., Kim, H. J., Jang, H. B., Lee, H. J. & Park, S. I. The Association between Alcohol Consumption and β-Cell Function and Insulin Sensitivity in Korean Population. *Int. J. Environ. Res. Public. Health. ***13** (11). 10.3390/ijerph13111133 (2016). 10.3390/ijerph13111133 PMC5129343 27854254

