# Supplementary material for: Association of ALDH2 rs671 Polymorphism with chronic kidney disease incidence in a population-based Korean cohort
Source: Sci Rep. 2026 Mar 15;16:13563. doi: 10.1038/s41598-026-43186-4 (PMC13121792; doi:10.1038/s41598-026-43186-4)
Supplement: Supplementary file 1 — Supplementary Material 1 [file 41598_2026_43186_MOESM1_ESM.docx]

**Association of *ALDH2* rs671 Polymorphism with Chronic Kidney Disease Incidence in a Population-Based Korean Cohort**

Hyun Jin Lee^1^, Jaekyung Noh^1^, Seunghwan Jeong^1^, Heeyeon Lee^3^, Haekyung Lee^1, 2^, Hyoungnae Kim^1, 2^, Jin Seok Jeon^1, 2^, Hyunjin Noh^1, 2^, and Soon Hyo Kwon^1, 2^

**^1^Division of Nephrology, Department of Internal Medicine, Soonchunhyang University Seoul Hospital,** 59 Daesagwan-ro, Yongsan-gu Seoul, 04401, Korea**.**

**^2^Hyonam Kidney Laboratory, Soonchunhyang University Seoul Hospital,** 59 Daesagwan-ro, Yongsan-gu Seoul, 04401, Korea**.**

**^3^Department of Biostatistics, Soonchunhyang University Seoul Hospital,** 59 Daesagwan-ro, Yongsan-gu Seoul, 04401, Korea**.**

**Corresponding Author:**

**Soon Hyo Kwon**

**Division of Nephrology, Department of Internal Medicine, Hyonam kidney Laboratory, Soonchunhyang University Seoul Hospital, 59 Daesagwan-ro, Yongsan-gu Seoul, 04401, Korea.**

Tel: +82-2-710-3274

E-mail: [ksoonhyo@schmc.ac.kr](mailto:ksoonhyo@schmc.ac.kr)

**Supplementary Table S1. Detailed Cox regression for overall cohort**

|  | Unadjusted | | Adjusted* | |
| --- | --- | --- | --- | --- |
|  | HR (95% CI) | *p value* | HR (95% CI) | *p value* |
| Age | 1.09 (1.08–1.10) | <0.001 | 1.08 (1.07–1.09) | <0.001 |
| Male | 0.78 (0.70–0.87) | <0.001 | 0.59 (0.46–0.75) | <0.001 |
| BMI | 1.07 (1.05–1.09) | <0.001 | 1.04 (1.02–1.06) | <0.001 |
| DM | 2.51 (2.11–2.99) | <0.001 | 1.80 (1.50–2.16) | <0.001 |
| HTN | 2.50 (2.20–2.84) | <0.001 | 1.52 (1.32–1.74) | <0.001 |
| Dyslipidemia | 0.92 (0.64–1.32) | 0.650 | 0.84 (0.58–1.21) | 0.350 |
| Smoking |  |  |  |  |
| Never | reference |  | reference |  |
| Former | 0.88 (0.74–1.03) | 0.113 | 1.08 (0.86–1.36) | 0.484 |
| Current | 0.82 (0.71–0.94) | 0.005 | 1.31 (1.06–1.62) | 0.011 |
| Physical activity (MVPA) | 1.15 (1.02–1.29) | 0.023 | 0.98 (0.87–1.11) | 0.761 |
| Hb | 1.00 (0.96–1.03) | 0.782 | 1.02 (0.96–1.08) | 0.557 |
| Income |  |  |  |  |
| Low | reference |  | reference |  |
| Intermediate | 0.61 (0.53–0.70) | <0.001 | 1.10 (0.95–1.29) | 0.201 |
| High | 0.54 (0.46–0.64) | <0.001 | 0.99 (0.81–1.20) | 0.884 |
| Education |  |  |  |  |
| Low | reference |  | reference |  |
| Middle | 0.62 (0.54–0.73) | <0.001 | 1.26 (1.07–1.49) | 0.006 |
| High | 0.51 (0.45–0.58) | <0.001 | 1.23 (1.04–1.45) | 0.017 |
| eGFR | 0.96 (0.95–0.96) | <0.001 | 0.96 (0.96–0.97) | <0.001 |

HR, hazard ratio; CI, confidence interval; BMI, body mass index; DM, diabetes mellitus; HTN, hypertension; HB, hemoglobin; MVPA, moderate-to-vigorous physical activity; EDU, education level; eGFR, estimated glomerular filtration rate.

* Adjusted for age, sex, BMI, DM, HTN, hyperlipidemia, smoking status, MVPA, Hb, income, education and eGFR.

**Supplementary Table S2. Sensitivity analysis: Cox proportional hazards models for incident eGFR decline**

|  | Unadjusted | | Adjusted* | |
| --- | --- | --- | --- | --- |
|  | HR (95% CI) | *p value* | HR (95% CI) | *p value* |
| *ALDH2* genotype, GG/(GA+AA) | 0.96 (0.84–1.10) | 0.541 | 1.07 (0.92–1.25) | 0.388 |
| Alcohol consumption,  (Moderate + High / None + Low) | 0.62 (0.54–0.72) | **<0.001** | 0.96 (0.65–1.42) | 0.851 |
| *ALDH2* genotype * alcohol consumption |  |  | 0.94 (0.62–1.41) | 0.755 |

*ALDH2*, aldehyde dehydrogenase 2; eGFR, estimated glomerular filtration rate; HR, hazard ratio; CI, confidence interval.

Incident eGFR decline was defined as estimated glomerular filtration rate <60 mL/min/1.73 m².

* Adjusted for age, sex, BMI, DM, HTN, hyperlipidemia, smoking status, MVPA, Hb, income, education and eGFR.

**Supplementary Table S3. Sensitivity analysis: Cox proportional hazards models for incident albuminuria**

|  | Unadjusted | | Adjusted* | |
| --- | --- | --- | --- | --- |
|  | HR (95% CI) | *p value* | HR (95% CI) | *p value* |
| *ALDH2* genotype, GG/(GA+AA) | 1.15 (0.89–1.50) | 0.289 | 1.18 (0.84–1.64) | 0.336 |
| Alcohol consumption,  (Moderate + High / None + Low) | 1.60 (1.26–2.02) | **<0.001** | 1.57 (0.89–2.78) | 0.119 |
| *ALDH2* genotype * alcohol consumption |  |  | 0.66 (0.36–1.21) | 0.178 |

*ALDH2*, aldehyde dehydrogenase 2; HR, hazard ratio; CI, confidence interval.

Incident albuminuria was defined as dipstick proteinuria ≥1+.

* Adjusted for age, sex, BMI, DM, HTN, hyperlipidemia, smoking status, MVPA, Hb, income, education and eGFR.

**Supplementary Table S4. Sex-stratified Cox proportional hazard models of incident CKD in men**

|  | Unadjusted | | Adjusted* | |
| --- | --- | --- | --- | --- |
|  | HR (95% CI) | *p value* | HR (95% CI) | *p value* |
| GG genotype |  |  |  |  |
| Alcohol consumption,  (Moderate + High / None + Low) | 0.82 (0.64–1.05) | 0.110 | 0.93 (0.72 – 1.20) | 0.573 |
| Age | 1.07 (1.06–1.09) | <0.001 | 1.07 (1.06–1.09) | <0.001 |
| BMI | 1.06 (1.02–1.10) | <0.001 | 1.05 (1.01–1.09) | 0.019 |
| DM | 2.40 (1.79–3.23) | <0.001 | 1.69 (1.25–2.29) | <0.001 |
| HTN | 2.71 (2.13–3.44) | <0.001 | 1.71(1.33–2.20) | <0.001 |
| GA+AA genotype |  |  |  |  |
| Alcohol consumption,  (Moderate + High / None + Low) | 1.15 (0.79–1.68) | 0.457 | 1.26 (0.84–1.88) | 0.267 |
| Age | 1.08(1.05–1.0) | <0.001 | 1.06 (1.03–1.09) | <0.001 |
| BMI | 1.05 (0.99–1.12) | 0.085 | 1.07(1.00–1.14) | 0.058 |
| DM | 2.60 (1.49–4.53) | 0.001 | 2.51 (1.40–4.50) | 0.002 |
| HTN | 310(2.09–4.62) | <0.001 | 1.54 (0.98–2.41) | 0.062 |

HR, hazard ratio; CI, confidence interval; BMI, body mass index; DM, diabetes mellitus; HTN, hypertension.

* Adjusted for age, sex, BMI, DM, HTN, hyperlipidemia, smoking status, MVPA, Hb, income, education and eGFR.

**Supplementary Table S5. Sex-stratified Cox proportional hazard models of incident CKD in women**

|  | Unadjusted | | Adjusted* | |
| --- | --- | --- | --- | --- |
|  | HR (95% CI) | *p value* | HR (95% CI) | *p value* |
| GG genotype |  |  |  |  |
| Alcohol consumption,  (Moderate + High / None + Low) | 0.57 (0.38–0.86) | 0.007 | 0.90 (0.59–1.38) | 0.642 |
| Age | 1.10 (1.09–1.12) | <0.001 | 1.09 (1.07–1.10) | <0.001 |
| BMI | 1.07 (1.04–1.10) | <0.001 | 1.05 (1.02–1.08) | 0.002 |
| DM | 2.61 (1.93–3.52) | <0.001 | 1.95 (1.43–2.65) | <0.001 |
| HTN | 2.20 (1.80–2.69) | <0.001 | 1.28(1.04–1.59) | 0.023 |
| GA+AA genotype |  |  |  |  |
| Alcohol consumption,  (Moderate + High / None + Low) | 0.8 (0.20–3.22) | 0.752 | 1.51 (0.37–6.14) | 0.565 |
| Age | 1.10 (1.09–1.12) | <0.001 | 1.08 (1.06–1.) | <0.001 |
| BMI | 1.05 (1.01–1.10) | 0.021 | 1.01(0.97–1.06) | 0.576 |
| DM | 2.87 (1.82–4.50) | 0.001 | 1.67 (1.03–2.70) | 0.036 |
| HTN | 2.56 (1.88–3.48) | <0.001 | 1.60 (1.14–2.24) | 0.007 |

HR, hazard ratio; CI, confidence interval; BMI, body mass index; DM, diabetes mellitus; HTN, hypertension.

* Adjusted for age, sex, BMI, DM, HTN, hyperlipidemia, smoking status, MVPA, Hb, income, education and eGFR.
